# Successful treatment of large hemoptysis and pseudoaneurysm of the pulmonary artery associated to oesophagomediastinal fistula with amphotericin B cholesterol sulfate complex: A case report

**DOI:** 10.1002/rcr2.70047

**Published:** 2024-10-14

**Authors:** Zhujun Chen, Jian He, Qin Huang, Peiqiang Liang, Liang Gong, Qiangzhong Pi

**Affiliations:** ^1^ Department of Respiratory and Critical Care Medicine The First Affiliated Hospital of Army Medical University (Southwest Hospital) Chongqing China

**Keywords:** case report, oesophagomediastinal fistula, pulmonary artery pseudoaneurysms, pulmonary fungal infection

## Abstract

Oesophagomediastinal fistula is uncommon. Oesophageal fistulas, may manifest as recurrent pneumonias. While pulmonary infections can lead to pulmonary artery pseudoaneurysms (PAPs), particularly in fungal infections. PAPs pose a rupture risk, potentially causing life‐threatening hemoptysis. We report a unique case of a 45‐year‐old male who presented with sudden cough, dyspnea, and hemoptysis. Bronchoscopy triggered massive hemoptysis, necessitating emergency embolization. Persistent hemoptysis prompted further imaging, revealing an aneurysmal dilation located next to the spine and infectious lesions, suggesting an oesophagomediastinal fistula. After initiating therapy with Amphotericin B Cholesterol Sulfate Complex and fistula closure, the patient's hemoptysis resolved, with imaging resolution of the PAP. Long‐term Voriconazole therapy ensured continued improvement. This case highlights the rarity and severity of such fistulas may be associated with fungal infections and PAPs, emphasizing the importance of prompt recognition, aggressive treatment for favourable outcomes.

## Graphical abstract

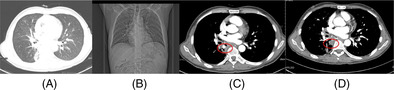

## INTRODUCTION

Oesophageal fistulas, albeit infrequent, are recognized precursors of pulmonary infections, a sequela that has been intimately linked to the development of PAPs, a rare and potentially lethal condition.^1^, ^2^, ^3^ These PAPs can escalate into life‐threatening scenarios, characterized by severe hemoptysis and asphyxia, presenting formidable therapeutic challenges.^3^ Notably, despite the well‐established correlation between pulmonary infections and PAPs, a conspicuous gap remains in the literature regarding the direct association between oesophagomediastinal fistulas and the occurrence of PAPs. PAPs pose a formidable challenge in medical practice, underscored by their high mortality rates and intricate management strategies.^4^ While pulmonary fungal infections, particularly aspergillosis, have been extensively documented as causes of PAPs, reports detailing mucormycosis as a contributing factor are scarce.^3^

In this report, we present an illustrative case of a patient diagnosed with pulmonary mucormycosis infection based on characteristic pulmonary imaging findings. Notably, the patient's PAP, which was associated with an oesophagomediastinal fistulas presented on chest imagings, was successfully resolved following targeted antifungal therapy with Amphotericin B Cholesterol Sulfate Complex. This case not only underscores the importance of early diagnosis and intervention but also provides invaluable insights for clinicians in navigating the diagnosis and treatment of this rare and complex condition.

## CASE REPORT

A 45‐year‐old male patient, without significant past medical history, initially presented with persistent and debilitating symptoms of dry cough and dyspnea for 1 month. He went to the local hospital. An initial chest CT scan conducted at an external facility revealed a mass in the right lower lung base segment, prompting further investigation. Subsequently, the patient underwent bronchoscopy for investigating the cause of hemoptysis, however, the procedure was complicated by a sudden onset of massive hemoptysis accompanied by a precipitous drop in blood pressure. To promptly address the life‐threatening haemorrhage, emergency embolization procedures were swiftly performed by physicians at the local hospital on the left and right main bronchi, the right intercostal artery, and the left main bronchial artery, due to the urgency of the situation and the unidentified bleeding vessel. These interventions partially mitigated the hemoptysis, enabling the patient's transfer to our institution for definitive management.

**FIGURE 1:**
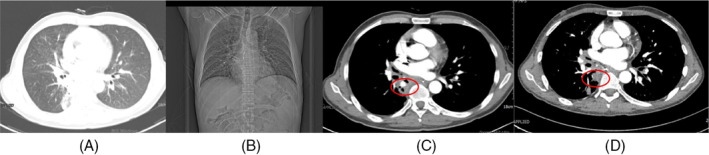
(A) Chest computed tomography (CT) lung window demonstrated a ‘mass’ before antifungal treatment; (B) the chest radiograph before antifungal treatment; (C) pseudo‐pulmonary artery aneurysm that appeared before antifungal treatment; (D) CT shows the disappearance of the pseudo‐pulmonary artery aneurysm after antifungal treatment.

**FIGURE 2:**
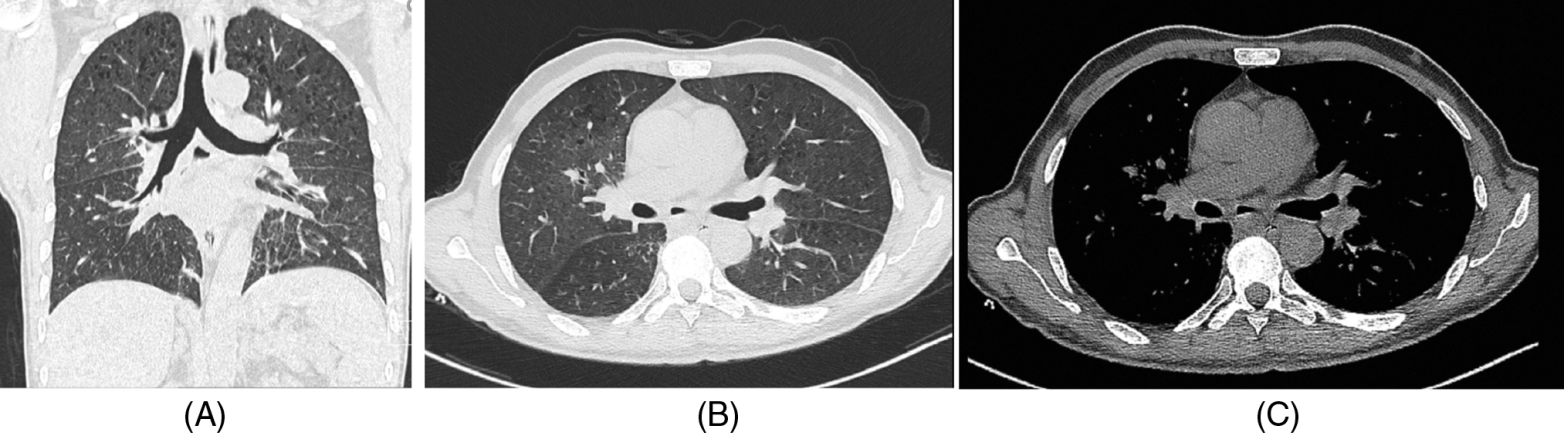
(A) Chest computed tomography longitudinal section, (B) lung window and (C) mediastinal window showed gas collections within the middle mediastinum below the tracheal carina.

**FIGURE 3:**
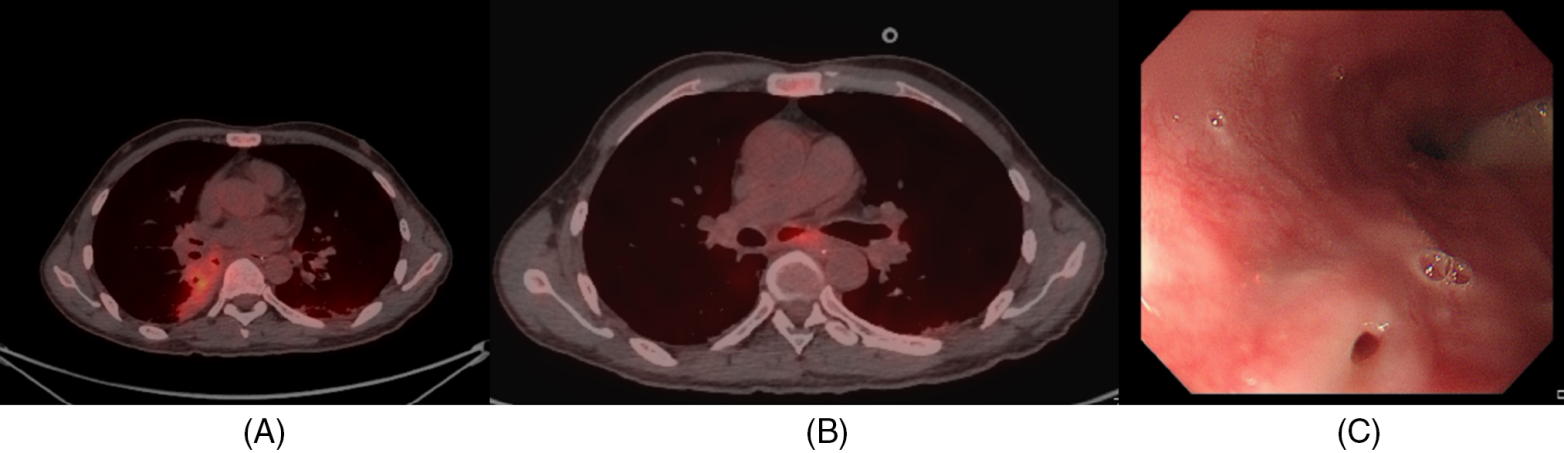
(A) positron emission tomography‐computed tomography before antifungal treatment demonstrated a patchy high‐density shadow in the right lower lobe with indistinct borders, accompanied by a cavity formation with an SUVmax of 6.7 and accompanied by multiple cystic lucent shadows; (B) a cystic lucent shadow was seen beneath the carina, with a slightly increased fluorodeoxyglucose uptake at the margins, yielding an SUVmax of 4.0, which was partially connected to the adjacent oesophagus, with multiple enlarged lymph nodes in the mediastinum, yielding; (C) gastroscopic image of the oesophageal mucosa (30 cm from the incisors) demonstrating an oesophagomediastinal fistula.

Upon admission to our department, he presented vital signs were recorded as follows: a temperature of 36.6°C, pulse of 89 beats per minute, breath of 18 breaths per minute, blood pressure of 115/61 mmHg. His arterial blood gas analysis indicated a pH of 7.46, an oxygen partial pressure of 83 mmHg, and a carbon dioxide partial pressure of 30 mmHg. A chest‐enhanced CT scan at our hospital revealed a PAP in the posterior segment of the right lower lung (Figure 1). Chest CT also revealed localized mediastinal gas collections, below the tracheal ridge (Figure 2), which suggests oesophagomediastinal fistula formation.^5^ To investigate whether the space‐occupying lesion is tumorous or infectious and clarify the oesophagomediastinal fistula diagnosis, a PET/CT scan (Figure 3A,B) was conducted which indicated an infectious aetiology surrounding the right PAP. On PET/CT scan, active glucose metabolism was observed around the air‐containing sac located beneath the carina, with the sac communicating with the oesophagus, which suggesting the presence of an oesophagomediastinal fistula accompanied by surrounding inflammation. Sputum culture, aspergillus antigen analysis and fungal D‐glucan detection of the patient were negative.

**FIGURE 4:**
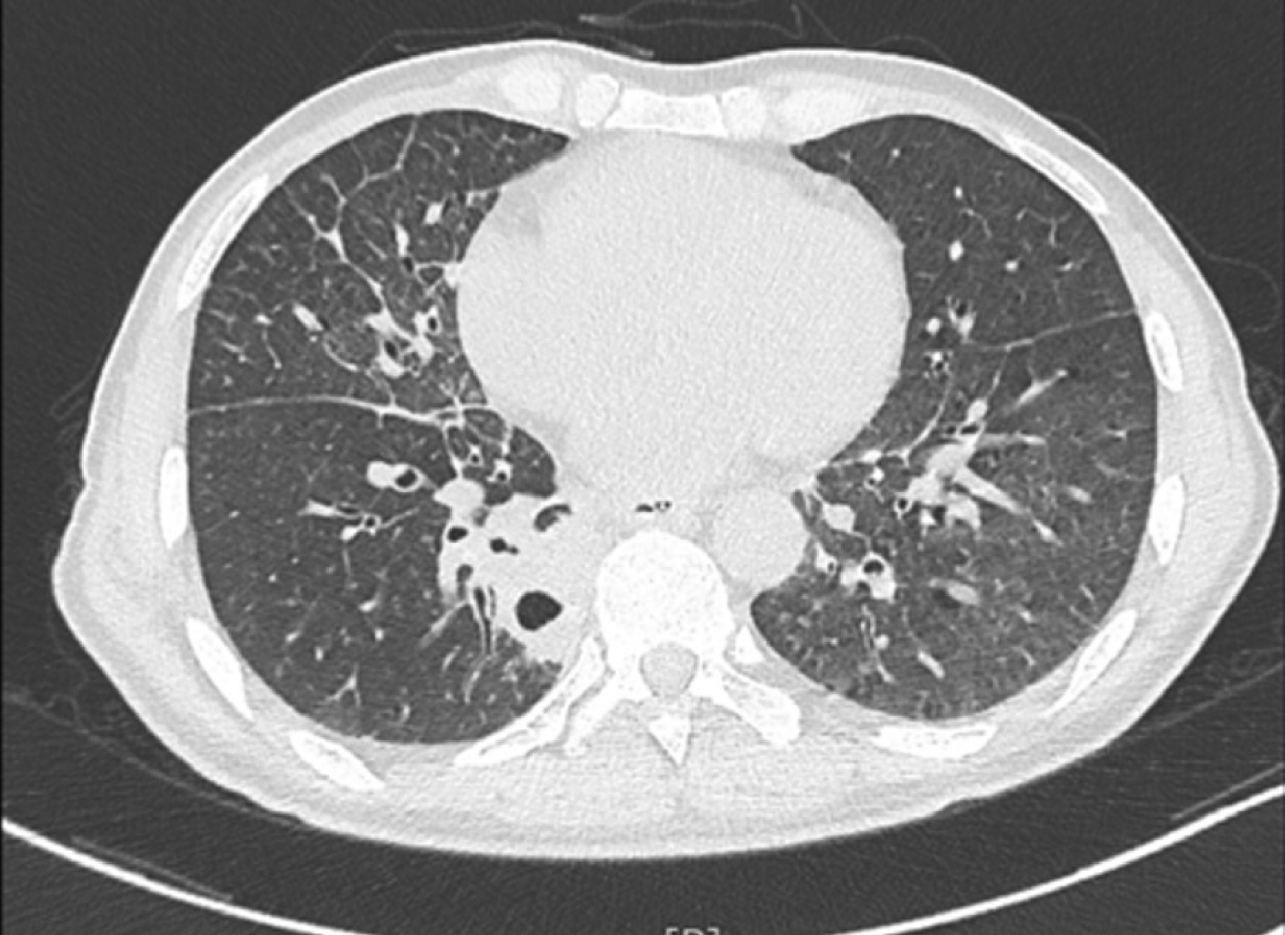
Computed tomography (CT) demonstrated cavitations, consolidations and mass, consistent with known CT manifestations of typical mucormycosis.

The challenge of this case is that the hemoptysis is considered to be related to infectious lesions in the lungs but the pathogen is unknown and the treatment effect is unsatisfactory. Therefore, it is difficult to determine the next treatment plan. While the patient had the symptoms including cough, dyspnea and hemoptysis, moreover, his chest CT showed “atypical” cavitations, consolidations and mass (Figure 4), consistent with known CT and clinical manifestations of typical mucormycosis.^6^, ^7^ Based on these comprehensive imaging findings, the radiologist made the initial clinical diagnosis as a mucormycosis infection, although the results of sputum culture, aspergillus antigen analysis, and fungal D‐glucan detection in the patient did not support the diagnosis of mucormycosis.

The patient clinically diagnosed as following: Pulmonary mycosis, pseudoaneurysm in the posterior segment of the right lower lung lobe, oesophagomediastinal fistula. The presence of an oesophagomediastinal fistula, was confirmed by gastroscopy (Figure 3C).

After meticulous discussion with the patient, a decision was made to initiate diagnostic antifungal therapy with intravenous administration of Amphotericin B Cholesterol Sulfate Complex (dose escalation from 50 to 200 mg daily) for 27 days. During the course of treatment, the patient's hemoptysis progressively resolved, and the lesion in the right lower lung demonstrated a notable reduction in size (Figure 1C), without obvious side effects.

Furthermore, after 18 days of hospitalization and significant alleviation of the patient's hemoptysis, we performed a gastroscopy. The examination revealed an oesophageal fistula with a diameter of approximately 0.5 cm in the oesophageal mucosa, located about 30 cm from the incisors (Figure 1E). Subsequently, an endoscopic oesophageal fistula closure procedure was carried out. Following the surgery, the patient did not experience any further hemoptysis.

Ultimately, the patient achieved a fully satisfying recovery and was successfully discharged from the hospital, highlighting the importance of prompt diagnosis, aggressive intervention, and targeted antifungal therapy in managing such complex and potentially life‐threatening conditions.

## DISCUSSION

The presented case highlights the occurrence of massive hemoptysis in a patient, subsequently diagnosed with a PAP in the right lower lobe of the lung and oesophagomediastinal fistula. PAPs represent a localized enlargement of a pulmonary artery segment, distinct from true aneurysms in their limited involvement of the arterial wall layers, primarily the media and adventitia. This structural distinction renders PAPs more prone to rupture, given the reduced supportive tissue, thereby posing a significant risk of haemorrhage.^8^ In particular, the rupture of a PAP frequently precipitates massive hemoptysis, an uncommon yet potentially life‐threatening event associated with an elevated mortality rate of up to 50%.^9^, ^10^

PAPs can arise congenitally or secondarily to various conditions, including vessel wall infections, trauma, neoplasia, pulmonary hypertension, and vasculitis.^11^ Notably, pulmonary mucormycosis (PM), particularly involving the pulmonary system, can elicit vessel wall inflammation, leading to progressive wall thinning and the formation of pseudoaneurysms. PM characterized by its vascular invasion, causing vessel wall inflammation, weakening, and eventual pseudoaneurysm formation.^12^, ^13^ Notably, the mortality rate for invasive mucormycosis ranges widely, from over 30%–50% to as high as 90% in disseminated cases.^8^ In severe cases, pulmonary fungal infections may extend to adjacent structures, such as the mediastinum, bronchoesophagus, pericardium, and chest wall.^11^, ^14^

The detection of PAPs on CT scans without intravenous contrast enhancement may be elusive, as they may mimic endobronchial lesions or lung masses. Thus, intravenous contrast enhancement is imperative for optimal visualization of pulmonary arteries and identification of any aneurysm formations.^15^ The development of PAP secondary to pulmonary mucormycosis (PM) is exceedingly rare, with diagnostic modalities such as computed tomography pulmonary angiography (CTPA) and bronchoscopy playing pivotal roles in confirmation.^16^ CT angiography emerges as a superior imaging technique for the identification of PAPs, offering detailed visualization of the pulmonary vasculature. CTPA not only facilitates accurate localization of the pseudoaneurysm and its feeding vessels but also informs treatment decisions.^16^ Our case also show the PET/CT facilitates the detection of PAPs.

For the treatment, endovascular occlusion of the offending vessel, using coils, plugs, or stents, represents the current standard of care for PAPs, aiming to halt blood flow to the pseudoaneurysm, thereby mitigating its expansion and rupture risk.^17^ In our case, the PAP, associated with an uncommon oesophagomediastinal fistulas, likely triggered by the fungal inflammatory response, which led to the pathological changes culminating in PAP development, in which endovascular occlusion outcome is not satisfactory.

However, the treatment for mucormycosis has been effective in our case. The diagnosis of massive hemoptysis secondary to pulmonary fungal infections often poses challenges, leading to delayed treatment and higher mortality.^7^ PM is not a common fungal infection, accounts for 11% of hemoptysis cases undergoing bronchial or pulmonary angiography and carries a poor prognosis due to diagnostic delays and limited treatment options.^14^ This case shows the localized mediastinal gas collections is signs that indicates oesophagomediastinal fistula and it underscores diagnosis of PM and the successful use of Amphotericin B Cholesterol Sulfate Complex in resolving the PAP caused by PM and oesophagomediastinal fistulas, highlighting the role of Amphotericin B Cholesterol Sulfate Complex as the cornerstone of mucormycosis‐therapy, with intravenous administration being the primary route. In cases where Amphotericin B Cholesterol Sulfate Complex is contraindicated or poorly tolerated, oral triazoles, such as posaconazole and isavuconazole, offer alternative therapeutic options.^18^

A primary limitation of our case is the results of sputum culture, aspergillus antigen analysis, and fungal D‐glucan detection in the patient did not support the diagnosis of mucormycosis and radiographic findings in pulmonary mucormycosis is nonspecific, mimicking those of pulmonary aspergillosis, However, the complete disappearance of the lung lesion after treatment confirms the diagnosis of PM.

In conclusion, oesophagomediastinal fistulas, especially when associated with pulmonary fungal infections and subsequent pulmonary artery aneurysms (PAPs), constitute rare and challenging clinical entities. In patients presenting with hemoptysis and imaging features suggestive of oesophageal fistulas, prompt consideration of fungal infection and the possibility of PAPs is imperative. To address these challenges, physicians must undertake comprehensive medical history assessments and imaging examination. Furthermore, meticulous monitoring of persistent and severe symptoms, coupled with detailed analysis of chest imaging and interdisciplinary collaboration, is crucial for achieving timely diagnosis and formulating effective treatment strategies.

## AUTHOR CONTRIBUTIONS

Zhujun Chen, Jian He and Qiangzhong Pi wrote the initial draft of manuscript and collected main data. Qing Huang and Peiqiang Liang managed the diagnosis and treatment. Liang Gong and Qiangzhong Pi conducted review and editing. All authors read and approved the final manuscript.

## CONFLICT OF INTEREST STATEMENT

None declared.

## ETHICS STATEMENT

The authors declare that appropriate written informed consent was obtained for the publication of this manuscript and accompanying images.

## Data Availability

Data sharing not applicable to this article as no datasets were generated or analysed during the current study.
